# Middle cerebral artery fenestration presenting with cerebral ischemia: a case report and review of the literature

**DOI:** 10.3389/fmed.2026.1879070

**Published:** 2026-06-09

**Authors:** Ying Bi, Zhifang Li, Wenxian Sun, Li Zou, Huagang Li, Bin Mei

**Affiliations:** 1 Department of Neurology, Zhongnan Hospital of Wuhan University, Wuhan, Hubei, China; 2 Hubei Provincial Clinical Research Center for Dementia and Cognitive Impairment, Zhongnan Hospital of Wuhan University, Wuhan, Hubei, China

**Keywords:** cerebral ischemia, fenestration, high-resolution MRI, middle cerebral artery, misdiagnosis, vessel wall imaging

## Abstract

**Background:**

Middle cerebral artery (MCA) fenestration is a rare vascular variant that can mimic stenosis. Its association with cerebral ischemia remains controversial.

**Method:**

We report the case of a 53-year-old man with a history of transient ischemic attack (TIA), initially misdiagnosed with left MCA occlusion, who presented with sleep disturbances due to fear of stroke recurrence. A definitive diagnosis of MCA fenestration was established by digital subtraction angiography (DSA) and high-resolution magnetic resonance vessel-wall imaging (HRMR-VWI). Perfusion-weighted imaging (PWI) was employed to evaluate the associated hemodynamic compromise. A literature review was conducted to identify all reported cases of MCA fenestration associated with cerebral ischemia.

**Results:**

A total of 19 cases (including ours) from 12 studies were identified. Patients were predominantly male, with ages ranging from 4 to 85 years. All fenestrations were located in the M1 segment. Slit-like morphology was observed in 73.7% of patients and was more frequent in acute ischemic stroke (AIS) than in TIA (91.7% vs. 42.9%). Eleven patients (57.9%) were definitively diagnosed via DSA. Mechanical thrombectomy was performed in six patients with concurrent MCA occlusion, yielding favorable functional outcomes in five (83.3%). Overall, 73.7% of patients achieved favorable outcomes at follow-up.

**Conclusion:**

MCA fenestration should be considered in the differential diagnosis for patients presenting with cerebral ischemia and M1 stenosis or occlusion, particularly in the absence of conventional vascular risk factors. A slit-like morphology may be associated with a higher risk of stroke. Multimodal imaging is essential to avoid misdiagnosis and guide appropriate clinical management.

## Introduction

Fenestration is a rare but well-known vascular variation of the cerebral arteries which refers to the division of an arterial segment into two parallel channels that subsequently rejoin distally, each channel maintaining its own endothelial and muscular layers. They most frequently occur in the anterior communicating artery, followed by the vertebrobasilar artery and the anterior cerebral artery (1). Fenestration of the middle cerebral artery (MCA) is an exceedingly rare anatomic variant, with reported estimates ranging from 0.17 to 4.4% depending on the imaging modality and study population (2–5).

Despite its low incidence, MCA fenestration assumes clinical importance for several reasons. First, its angiographic appearance may closely mimic that of acquired vascular pathologies such as atherosclerotic stenosis or arterial dissection, posing a significant diagnostic challenge and potentially leading to inappropriate management. Second, the hemodynamic consequences of this anatomical variant remain incompletely understood. Although some authors have proposed that fenestration may create local flow disturbances that predispose to thrombus formation and distal embolization, the relationship between MCA fenestration and cerebral ischemia has not been systematically characterized (6).

In this study, we report a case of MCA fenestration, discuss its clinical and imaging features, and highlight its diagnostic challenges. We also present a systematic literature review of MCA fenestration associated with cerebral ischemia-the largest series to date, comprising 19 cases-to characterize the clinical, radiological, and prognostic features of this condition. Through this study, we aim to: (1) emphasize the value of high-resolution vessel wall MRI (HRMR-VWI) in differentiating fenestration from atherosclerotic stenosis or dissection by demonstrating normal vessel wall characteristics; (2) propose perfusion-weighted imaging (PWI) as a valuable tool for hemodynamic assessment and risk stratification for stroke prevention; and (3) provide an evidence-based framework to guide clinical management of this uncommon but clinically significant vascular variant.

## Case presentation

A 53-year-old man presented to the neuropsychology department of our hospital with an 8-year history of sleep disturbance. His medical history included type 2 diabetes mellitus, managed with metformin hydrochloride and empagliflozin for the past 6 years. On further questioning, he recalled a transient episode of speech disturbance 8 years ago, characterized by word-finding difficulty that resolved completely within 1 h. At that time, he was diagnosed elsewhere with cerebral ischemia secondary to left MCA occlusion based on neuroimaging findings (exact modality unspecified in retrospective recall). He was subsequently initiated on aspirin and statin therapy for secondary stroke prevention. Shortly after this diagnosis, the patient developed persistent sleep disturbances, which he attributed to anxiety and persistent fear of symptom recurrence. Despite reassurance and symptomatic treatment, these sleep difficulties persisted, ultimately prompting referral to our neuropsychology department for further evaluation.

Upon admission, a systemic neurological examination revealed no definitive positive findings. Magnetic resonance imaging (MRI) with three-dimensional time-of-flight (3D-TOF) angiography demonstrated an apparent severe stenosis to occlusion of the left M1 segment (Figure 1). Given this finding and the patient’s prior history of cerebral ischemia, he was referred to our department for evaluation for MCA recanalization.

**Figure 1:**
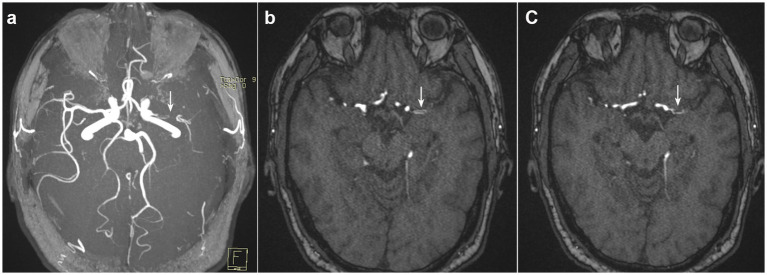
Initial misdiagnosis on MR angiography. **(a)** Three-dimensional time-of-flight (TOF) MRA shows an apparent occlusion of the left M1 segment (arrow). **(b,c)** Source images from TOF-MRA reveal a focal intraluminal filling defect (**b,c**, arrows).

To further evaluate the degree of stenosis and determine the underlying etiology, digital subtraction angiography (DSA) and high-resolution magnetic resonance vessel wall imaging (HRMR-VWI) were performed. DSA of the left internal carotid artery revealed a bifurcation of the left M1 segment into two parallel channels, forming a “double-barrel” appearance, which re-converge after a course of 9.19 mm (Figure 2). Contrast medium flowed uniformly within both channels, with no evidence of significant stasis or filling defects. 3D rotational reconstruction confirmed preserved distal vascular morphology. HRMR-VWI further characterized the lesion, demonstrating T1 isointensity within both lumens, with no abnormal vessel wall enhancement, thickening, or irregularity (Figure 3). Collectively, these findings were diagnostic of MCA fenestration involving the M1 segment, definitively excluding the initial suspicion of occlusive disease.

**Figure 2:**
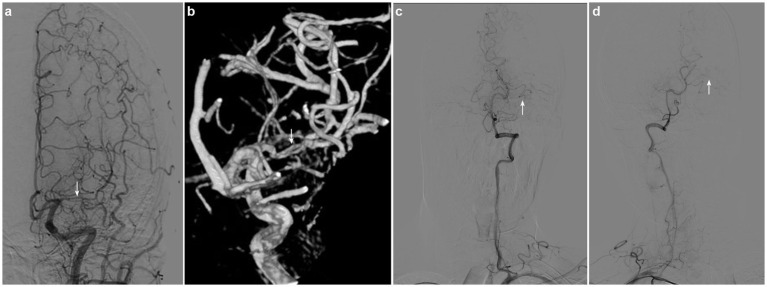
Definitive diagnosis of MCA fenestration by digital subtraction angiography (DSA). **(a)** Left internal carotid artery (ICA) angiogram shows a slit-like fenestration in the mid-M1 segment of the left MCA, with two parallel channels reconstituting distally (arrow). **(b)** Three-dimensional rotational reconstruction confirms preserved distal vascular morphology without stenosis or occlusion. **(c,d)** Bilateral vertebral artery angiography demonstrates leptomeningeal collateral supply from the left posterior cerebral artery to the left MCA territory (arrows), indicating compensatory hemodynamic adaptation.

**Figure 3:**
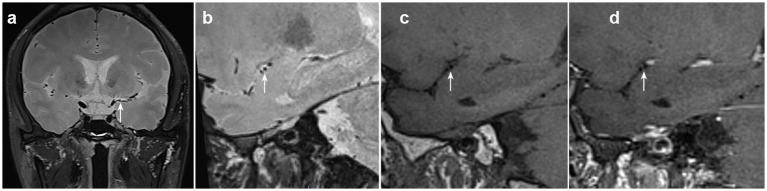
High-resolution vessel wall MRI (HRMR-VWI) features. **(a)** Coronal black-blood T1-weighted imaging shows a focal intraluminal filling defect in the left M1 segment. **(b)** Sagittal view demonstrates the vessel dividing into two distinct lumina. **(c)** Pre-contrast and **(d)** post-contrast T1-weighted imaging reveal a thin, slit-like, iso-intense intraluminal flap (arrows) separating the vessel into two channels, with no abnormal vessel wall enhancement, excluding atherosclerosis or dissection.

To assess whether the fenestration affected cerebral blood flow perfusion, perfusion-weighted imaging (PWI) was performed. Quantitative analysis revealed no significant interhemispheric differences in cerebral blood flow (CBF) or cerebral blood volume (CBV) (Figure 4). However, the mean transit time (MTT) and time to peak (TTP) perfusion map showed a slight prolongation in the left MCA fenestration region, suggesting mild hemodynamic compromise in this region. This was corroborated by DSA findings, which demonstrated leptomeningeal collateral flow from the left posterior cerebral artery to the left MCA territory (Figure 2), indicating compensatory circulation in response to reduced antegrade flow through the fenestrated segment (Figure 2).

**Figure 4:**
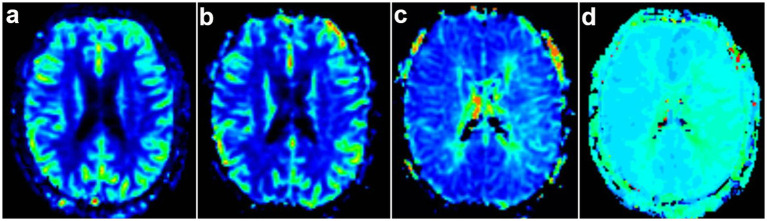
Perfusion-weighted imaging (PWI) findings. Quantitative analysis shows preserved cerebral blood flow (CBF) **(a)** and cerebral blood volume (CBV) **(b)** in the left MCA territory, but prolonged mean transit time (MTT) **(c)** and time to peak (TTP) **(d)**, indicating mild hemodynamic compromise attributable to the fenestration.

The episode of word-finding difficulty 8 years prior was retrospectively diagnosed as a TIA attributable to the MCA fenestration. The patient was advised to continue aspirin and atorvastatin for secondary stroke prevention, along with strict glycemic control. In the absence of hemodynamically significant stenosis or an indication for surgical intervention, the definitive diagnosis of fenestration-rather than occlusive disease-markedly alleviated the patient’s anxiety and associated sleep disturbances. At the 12-month follow-up, the patient remained free of recurrent cerebrovascular events and reported partial improvement in sleep quality.

## Literature review

### Search strategy and study selection

To define the clinical spectrum and diagnostic approaches for MCA fenestration and to elucidate its association with ischemic stroke risk, a comprehensive literature search was conducted without date restrictions (up to 23 October 2025) across Embase, PubMed, and Web of Science databases. The search strategy combined subject headings and free-text terms related to “fenestration,” “middle cerebral artery”, and “cerebral ischemia.” After excluding duplicates and non-English articles, 19 cases of MCA fenestration associated with cerebral ischemia, including the present case, were retained for retrospective analysis.

### Demographic and anatomical characteristics

The clinical, radiological, and prognostic characteristics of the 19 identified cases were summarized in Table 1. The cohort consisted of 13 male and 6 female patients (male-to-female ratio > 2:1), with ages ranging from 4 to 85 years (median, 65 years) (4, 7–17).

**Table 1:** Cases of MCA fenestration associated with cerebral ischemia described in the literature.

NO.	Sex	Age	Clinical presentation	NIHSS score	Risk factors	Infarction location	Fenestration location	Fenestration morphology	Diagnostic modality	Type of cerebral ischemia	Surgical intervention	Medication	Outcome	References
1	Man	32	Sudden onset of a thunderclap headache	0	No risk factors	Right frontal lobe	Right, M1 proximal	Small slit-like	CTA、DSA	AIS, SAH	None	No anticoagulant or thrombolytic treatment	Discharged home 1 week later, with no neurological deficit	(4)
2	Man	85	NA	NA	NA	NA	Left, M1 proximal	Small slit-like	MRA	AIS	NA	NA	NA	(7)
3	Man	64	NA	NA	NA	NA	Right, M1 proximal	Small slit-like	MRA	AIS	NA	NA	NA	(7)
4	Woman	82	Recurrent TIA	NA	NA	NA	Right, M1 proximal	Irregular morphology	DSA	TIA	NA	NA	NA	(8)
5	Woman	54	An episode of transient left hand clumsiness and dysarthria lasting “a few minutes”	NA	HTN, DM, dyslipidemia, obesity, smoking	No infarction	Right, M1 intermediate	A large, convex, lens-like fenestration	3D volume rendering of m MRA data and virtual endoscopy	TIA	None	Specific medications	NA	(9)
6	Man	80	Dysarthria	NA	HTN, prior IHD	No infarction	Right, M1 proximal	Small slit-like	3D volume rendering of m MRA data and virtual endoscopy	TIA	None	Intravenous heparin during the acute phase of stroke, and antiplatelet therapy was administered for a longer duration	Remained stroke-free during the 475-day follow-up	(17)
7	Woman	74	Right-sided hemiparesis (MRC grade IV), hypesthesia, dysarthria, and right central type facial palsy	NA	NA	Left periventricular white matter	Left, M1 intermediate	Small slit-like	3D volume rendering of m MRA data and virtual endoscopy	AIS	None	Intravenous heparin during the acute phase of stroke, and antiplatelet therapy was administered for a longer duration	Remained stroke-free during the 475-day follow-up	(17)
8	Woman	68	Transient dysarthria and left hand clumsiness	NA	DM	No infarction	Right, M1 proximal	Duplication of arteries	3D volume rendering of m MRA data and virtual endoscopy	TIA	None	Intravenous heparin during the acute phase of stroke, and antiplatelet therapy was administered for a longer duration	Remained stroke-free during the 475-day follow-up	(17)
9	Man	57	Dysarthria	NA	DM, dyslipidemia	No infarction	Right, M1 intermediate	Small slit-like	3D volume rendering of m MRA data and virtual endoscopy	TIA	None	Intravenous heparin during the acute phase of stroke, and antiplatelet therapy was administered for a longer duration	Remained stroke-free during the 475-day follow-up	(17)
10	Man	51	Recurrent symptoms of dysarthria and left-sided facial palsy	NA	Dyslipidemia, ex-smoker	No infarction	Right, M1 proximal	Small slit-like	3D volume rendering of m MRA data and virtual endoscopy	TIA	None	Not treated with intravenous heparin or antiplatelet therapy	Remained stroke-free during the 475-day follow-up	(17)
11	Man	78	Left hemiplegia and coma	NA	NA	Right putamen	Right, M1 proximal	Small slit-like	Follow-up DSA at 7 days post-intravascular thrombolysis	AIS	Urolinase infusion directly into the right MCA		Discharged 14 days later with no neuroligical deficits	(10)
12	Man	73	Acute left hemiparesis	10	Implanted epicardial pacemaker for cardiac arrhythmias	Right putamen	Right, M1 proximal	Small slit-like	Postoperative internal cerebral angiography DSA	AIS	MT	NA	Discharged home with a mRS score of 1 on the 7th day from onset.	(11)
13	Man	70s	Sudden disturbances in consciousness, right hemiplegia, and aphasia	26	NA	Left striatum	Left, M1 proximal	Small slit-like	DSA	AIS	MT, ADAPT	NA	Right hemiparesis improved; sensory aphasia persisted. The patient was transferred to a rehabilitation hospital on the 38th day after MT	(12)
14	Man	60s	Right hemiparesis and aphasia	22	Atrial fibrillation	Left basal ganglia and insula	Left, M1 intermediate	Small slit-like	DSA after EVT	AIS	MT, ADAPT	Intravenous recombinant tissue-type plasminogen activator	Paralysis improved, mild aphasia persisted and discharged home on the 13th day after MT	(12)
15	Woman	65	Sudden onset of left-limb weakness	18	Heart valve replacement and atrial fibrillation	NA	Left, M1 intermediate	Small slit-like	DSA after EVT	AIS	MT	NA	Hospitalized for rehabilitation with mRS 5 at the 3-month follow-up	(13)
16	Woman	49	Dysarthria and left hemiparesis MRC grade II	8	No risk factors	Right basal ganglia and anterior temporal lobe	Right, M1 intermediate	Small slit-like	DSA after EVT	AIS	MT	Intravenous recombinant tissue plasminogen activator	Recovered (mRS of 0) and discharged on the 28th day	(14)
17	Man	65	Right hemiplegia, dysarthria, and aphasia	12	Ischemic stroke history and homocysteinemia	Left hemicerebrum area	Left, M1 distal	Duplication of arteries	DSA after EVT, VW-MRI	AIS	MT, SWIM	NA	Recovered and remained troke-free and with a mRS score of 1 at 6-months follow-up	(15)
18	Boy	4	Right-sided hemiparesis ([mRS] 1/5), drowsiness, facial asymmetry, and weak gag reflex	NA	No risk factors	Left lentiform nucleus, caudate, and frontal subcortical hemisphere	Left, M1 intermediate	Small slit-like	VW-MRI, DSA	AIS	None	Ecosprin, physiotherapy, and speech therapy	Gradually improved and discharged	(16)
Present case	Man	53	A transient episode of word-finding difficulty	NA	DM	No infarction	Left, M1 intermediate	Duplication of arteries	DSA, VW-MRI	TIA	None	Aspirin and atorvastatin	Free of recurrent stroke at the 6-month follow-up.	

DM, diabetes mellitus; IHD, ischemic heart disease; HTN, hypertension; MT, Mechanical Thrombectomy; ADAPT, a direct aspiration first pass technique; SWIM, Stent retriever partially retracted with intermediate catheter for mechanical thrombectomy; mRS, Modified Rankin Scale; MRC, medical research council; SAH, subarachnoid hemorrhage; AIS, acute ischemic stroke; TIA, transient ischemic attack; 3D volume rendering of m MRA, Three-dimensional volume rendering of brain magnetic resonance angiography; HRMR-VWI, high-resolution magnetic resonance vessel-wall imaging; NA, not available.

All fenestrations were located in the M1 segment of the MCA, involving the proximal segment in 10 patients (52.6%), the mid-segment in 8 (42.1%), and the terminal segment in 1 (5.3%). A slight right-sided predominance was observed, with 11 fenestrations (57.9%) in the right MCA. Morphologically, the most common was the slit-like type, observed in 14 patients (73.7%), followed by arterial duplication in 3 (15.8%), convex lens-shaped type in 1 (5.3%), and irregular type in 1 (5.3%).

### Clinical presentations

Acute ischemic stroke (AIS) was the presenting manifestation in 12 patients (63.2%), while TIA occurred in 7 patients (36.8%). Common presenting symptoms included hemiparesis or limb weakness (*n* = 11), speech disturbance (aphasia or dysarthria) (*n* = 11) and altered consciousness (*n* = 3). Hemiparesis was observed in at least nine patients (81.8%) with AIS. Among the seven TIA patients, six (85.7%) exhibited language impairment, whereas the clinical symptoms of the remaining patient were not described in detail. Of the three patients with impaired consciousness, two comatose patients presented with MCA thrombosis, and the lethargic patient was a 4-year-old boy. Additionally, one patient presented with headache and was found to have subarachnoid hemorrhage within an area of fenestration, accompanied by AIS.

Notably, slit-like morphology was more prevalent in patients presenting with AIS compared to those with TIA (11/12, 91.7% vs. 3/7, 42.9%), suggesting that this morphological subtype may confer a higher thromboembolic risk. In AIS patients, infarcts were predominantly located in the basal ganglia/putamen (*n* = 6), followed by the frontal lobe (*n* = 2), periventricular white matter (*n* = 2), and insula (*n* = 1).

### Vascular risk factors

Traditional vascular risk factors were present in 8 (42.1%) patients, including hypertension (*n* = 2), diabetes mellitus (*n* = 4), atrial fibrillation (*n* = 2), dyslipidemia (*n* = 3), and smoking (*n* = 2). Notably, at least three patients had no identifiable risk factors, suggesting that fenestration itself may be an independent contributor to cerebral ischemia.

### Diagnostic modalities

Definitive diagnosis was established primarily by DSA, the gold standard modality, which was performed in 11 patients (57.9%). In seven of these patients, DSA was performed after thrombectomy or thrombolysis; they had initially been diagnosed with stenosis or occlusion, with fenestration identified only on post-procedural imaging. Other modalities included three-dimensional MRA with volume rendering (*n* = 5, 26.3%), HRMR-VWI (*n* = 3, 15.8%), and CTA (*n* = 1, 5.3%). HRMR-VWI has emerged as a valuable adjunctive tool, enabling differentiation from atherosclerotic stenosis or dissection by demonstrating normal vessel wall characteristics. Perfusion-weighted imaging (PWI) was performed only in our case to assess hemodynamic status.

### Treatment and outcomes

Overall, 14 patients (73.7%) achieved favorable outcomes (modified Rankin Scale [mRS] 0–2), 1 patient (5.3%) had severe disability (mRS 5), and 4 patients (21.1%) had no reported outcome data. No deaths occurred. Among the 12 AIS patients, 6 underwent mechanical thrombectomy, with baseline NIHSS scores ranging from 8 to 26. Of these, 5 patients (83.3%) achieved favorable functional outcomes (mRS 0–2), while the remaining patient had a poor outcome (mRS 5). Additional treatments included intra-arterial urokinase (*n* = 1) and intravenous rt-PA (*n* = 2). All TIA patients were managed conservatively with antiplatelet or anticoagulant therapy and generally had good outcomes. Three patients (1 AIS, 2 TIA) received no specific treatment.

## Discussion

MCA fenestration is a rare congenital vascular anomaly that has been increasingly recognized as a potential cause of cerebral ischemia. We report a unique case of MCA fenestration in a patient who was presented with a TIA but was initially misdiagnosed with MCA occlusion. This misdiagnosis led to years of anxiety and sleep disturbances until the correct diagnosis was ultimately established at our institution. We also systematically review the literature to characterize the clinical, radiological, and prognostic features of this condition. Our review, the largest to date comprising 19 cases, reveals several key findings: (1) all the fenestrations were located in the M1 segment, predominantly involving the proximal and mid portions; (2) slit-like morphology was the most common type (73.7%) and was associated with AIS (91.7% vs. 42.9% in TIA); (3) multimodal imaging, particularly DSA and HRMR-VWI, is essential for accurate diagnosis; and (4) most patients achieve favorable outcomes with appropriate management.

Fenestration is an embryonic variant during the development of cerebral vascular system. MCA fenestration is a rare congenital anomaly which is incidentally discovered during autopsy or angiography (18, 19). It is widely accepted that this anomaly results from incomplete or abnormal fusion of the primitive MCA arterial network between embryonic days 30 and 40. In this study, the right side was most frequently involved (57.9%), in line with prior series reporting rates of 50–80% (7, 17).

M1-segment fenestration is the most common form of MCA fenestration and can be subdivided into proximal, middle, and distal subtypes. In our cohort, all fenestrations were located in the M1 segment, with a predilection for the proximal portion (52.6%), followed by the mid- (42.1%) and distal segments (5.3%). Among the ten fenestrations located in the proximal M1 segment, the majority (8/10, 80%) were specifically of the slit-like morphological type, in line with prior reports describing proximal M1 fenestrations as typically small and slit-like (20, 21). The proximal segment, being the site of the most complex hemodynamic interactions during development, may be particularly susceptible to incomplete fusion. The rarity of distal segment involvement (only one case) may reflect the sequence of arterial twig fusion during embryonic development.

Cerebral arterial fenestration can closely mimic dissection or stenosis, posing a significant diagnostic challenge (22). MRA and computed tomography angiography (CTA) are the most commonly used imaging examinations for detecting vascular abnormalities. Although time-of-flight magnetic resonance angiography (TOF MRA) avoids ionizing radiation, its limited spatial resolution hampers evaluation of small-caliber arteries, as illustrated in the present case. Despite high sensitivity and specificity, CTA may still miss fenestrations. High-resolution vessel wall MRI (HRMR-VWI) and digital subtraction angiography (DSA) are therefore essential for accurate diagnosis. On HRMR-VWI, an MCA fenestration may appear as a thin, iso-intense structure within the lumen in T1-weighted images that divides the vessel into two separate channels, with or without contrast enhancement, features that distinguish it from atherosclerotic plaque or dissection (15, 16). DSA shows a linear filling defect that divides the lumen into two parallel compartments. With exceptional spatial resolution and dynamic flow imaging, DSA remains the gold standard for diagnosing intracranial arterial fenestrations. In particular, three-dimensional DSA affords unrestricted, multi-angle interrogation of cerebrovascular lesions, significantly enhancing diagnostic yield.

The diagnostic pitfalls are illustrated by two reported cases. A 66-year-old woman with acute dizziness was initially diagnosed with vertebral artery V1-segment dissection on the basis of CTA showing a double-lumen sign and intimal flap; however, subsequent DSA demonstrated a congenital V1 fenestration. Similarly, a 4-year-old boy presented with AIS manifesting as right hemiplegia. CTA revealed a filling defect in the left M1 middle cerebral artery segment; comprehensive workup for vasculitis, thrombophilia, and cardioembolic sources was negative. HRMR-VWI demonstrated T1-isointense, T2-hypointense dual lumina within the M1 segment without enhancement, intramural hematoma, or plaque-findings diagnostic of congenital MCA fenestration. DSA confirmed an ovoid mid-M1 filling defect without false lumen or contrast stasis, definitively establishing the diagnosis. In both cases, accurate diagnosis averted inappropriate anticoagulation or unnecessary endovascular intervention, eliminated redundant follow-up imaging, and relieved patients of psychological burden. These cases underscore the value of HRMR-VWI in resolving diagnostic uncertainty. Based on our experience and the literature, we recommend the use of HRMR-VWI for the detection of intracranial arterial fenestration in the following settings: (1) young patients with ischemic stroke lacking conventional vascular risk factors; (2) cases where conventional imaging shows fenestration with concomitant luminal stenosis, requiring differentiation among atherosclerosis, dissection, and vasculitis; and (3) patients presenting with recurrent or atypical territorial infarcts within the fenestration’s vascular distribution. Furthermore, for a more comprehensive evaluation of cerebrovascular stenosis, HRMR-VWI is similarly indicated in the following scenarios: (1) young patients (<50 years) without traditional risk factors; (2) stenosis with atypical morphology (e.g., long-segment, concentric, or bilateral symmetrical lesions); (3) clinical suspicion of intracranial arterial dissection or central nervous system vasculitis; (4) symptomatic intracranial atherosclerosis requiring assessment of plaque vulnerability (e.g., intraplaque hemorrhage, contrast enhancement, or positive remodeling); and (5) pre-interventional evaluation for intracranial arterial intervention. The underlying principle unifying these indications is that HR-VWI should be pursued in cases where luminal imaging alone is insufficient to definitively establish the etiology, and where the acquisition of additional information is likely to bear directly on diagnostic or therapeutic decisions.

MCA fenestration appears to increase the risk of cerebral ischemia through hemodynamic disturbances. Horia Pleş proposed that fenestration creates a functional flow-division stenosis, precipitating turbulence and impaired distal perfusion (9). Three-dimensional MRA has demonstrated pronounced asymmetry between the upper and lower limbs of the fenestration, supporting this hypothesis. In the present case, PWI revealed hypoperfusion within the cortical territory supplied by the fenestrated segment, while DSA disclosed that the left hemisphere depended on collateral flow from the posterior circulation. Both findings corroborated a focal hemodynamic disturbance attributable to the vascular anomaly. Despite these subclinical perfusion abnormalities, the patient’s long-term clinical stability suggests that the congenital variant was physiologically well-tolerated, likely due to robust collateral compensation. We therefore postulate that the index TIA arose from a confluence of factors: local turbulent flow predisposing to microembolus formation, coupled with a transient, perhaps stress-induced, failure of these collateral pathways to maintain adequate perfusion.

A key finding of our review is the strong association between morphological subtype and stroke risk. Slit-like fenestrations were markedly overrepresented in AIS (11/12, 91.7%) compared with TIA (3/7, 42.9%). This predilection for thromboembolic events suggests that the specific geometry of a fenestration may be a more critical determinant of clinical presentation than the mere presence of the anomaly itself. We hypothesize that the slit-like morphology represents an extreme phenotype of this vascular variant, characterized by one or both channels being hypoplastic or exhibiting an unfavorable angle of origin. Such a narrow, often asymmetrical, geometry is hemodynamically disadvantaged, predisposing to flow disturbance (23). Computational fluid dynamics (CFD) studies on cerebral aneurysms have established that regions of low wall shear stress (WSS) and complex, turbulent flow patterns are pro-atherogenic and pro-thrombotic (24). By analogy, we propose that the slit-like fenestration creates a local hemodynamic milieu characterized by flow deceleration, stasis, and oscillatory shear stress within its limbs or at the distal re-convergence point. These conditions not only predispose to in-situ thrombus formation but may also facilitate the aggregation of platelets and microemboli, which can then be flushed distally into the parental MCA territory. The fact that the majority of infarcts in our AIS cohort were located in the basal ganglia and subcortical regions, territories directly downstream of the M1 segment, strongly supports this mechanism of distal embolization originating from the fenestration site. This observation parallels findings in the vertebrobasilar circulation, where fenestrations with specific configurations-such as a left-deviated course or stenotic limb terminals-have been associated with an increased risk of ischemia (25). Whether the caliber of the individual limbs, the longitudinal extent, or the precise location of an intracranial arterial fenestration independently modulates cerebral ischemic risk remains to be established in adequately powered, prospective studies.

In our cohort, five of six patients with acute MCA occlusion within a fenestrated segment who underwent emergency mechanical thrombectomy achieved favorable functional outcomes. This 83.3% favorable rate compares favorably with outcomes in standard MCA occlusions, suggesting that timely intervention in this subgroup is effective. The direct aspiration first-pass technique (ADAPT) may be particularly suitable, as it generates less mechanical stress on the vessel wall than stent-retriever thrombectomy, potentially reducing the risk of vascular injury in this anatomically variant setting (12). Prompt recognition of fenestration upon encountering an atypically configured M1 occlusion on baseline imaging or during mechanical thrombectomy can meaningfully reduce procedural complications.

For MCA fenestration patients with cerebral ischemia, long-term antiplatelet therapy appears safe and effective. Of the cohort, six patients who received antiplatelet therapy for secondary stroke prevention remained free of recurrent cerebrovascular events and hemorrhagic complications throughout extended follow-up. The clinical value of hemodynamic assessment is increasingly recognized. A recent multicenter, retrospective, international study demonstrated that hypoperfusion mismatch is associated with a significantly elevated risk of recurrent ischemic stroke (26). Data from the CMOSS study additionally affirm that compromised distal perfusion heralds an increased hazard of stroke recurrence (27). These findings underscore the importance of reperfusion imaging for stroke-risk stratification. When MCA fenestration is detected, comprehensive whole-brain perfusion imaging should be obtained to quantify any hemodynamic impairment, permitting precise risk assessment and informing evidence-based decisions for primary prevention.

Several limitations of this study should be acknowledged. First, A limitation of our case is that DWI, T2, and FLAIR sequences were not performed due to the absence of neurological deficit symptoms, thereby precluding direct assessment of acute cerebral infarction or white matter lesions. Nonetheless, the absence of corresponding findings on high-resolution MRI (Supplementary Figure S1), together with cerebral perfusion data that did not support AIS, is collectively consistent with the absence of acute cerebral infarction in this patient. To mitigate this limitation, we will monitor for potential ischemic changes on T2 and FLAIR sequences during future follow-up. We suggest that subsequent prospective studies incorporate these sequences for further validation. Second, the small number of identified cases (*n* = 19) reflects the rarity of this condition but limits the generalizability of our findings. Third, publication bias may have influenced the reported clinical and radiological features, as cases with atypical presentations or poor outcomes may be overrepresented. Fourth, the absence of long-term follow-up data in some cases limits our understanding of the natural history and recurrent stroke risk. Fifth, medication data were incomplete for several patients, potentially limiting the depth of our treatment analysis. Finally, as a single-center case report combined with a literature review, our findings should be interpreted with caution and confirmed in larger, prospective multicenter studies.

## Conclusion

MCA fenestration is an extremely rare vascular anomaly that is easily overlooked. Clinicians should maintain a high index of suspicion for this rare anomaly in patients with M1 segment abnormalities, particularly those lacking traditional vascular risk factors. Slit-like morphology identifies a subgroup at increased thromboembolic risk and may serve as an imaging biomarker for stroke risk stratification. Multimodal imaging-combining DSA for definitive diagnosis, HRMR-VWI for vessel wall characterization, and PWI for hemodynamic assessment is essential for accurate diagnosis and optimal management. Future multicenter registries with standardized imaging protocols and long-term follow-up are needed to further elucidate the natural history, refine risk stratification, and establish evidence-based treatment guidelines for this uncommon but clinically significant condition.

## Glossary

MCA: Middle cerebral artery
TIA: Transient ischemic attack
DSA: Digital subtraction angiography
HRMR-VWI: High-resolution magnetic resonance vessel-wall imaging
PWI: Perfusion-weighted imaging
MRI: Magnetic resonance imaging
ICA: Internal carotid artery
CBF: Cerebral blood flow
CBV: Cerebral blood volume
MTT: Mean transit time
TTP: Time to peak
CTA: Computed tomography angiography
TOF MRA: Time-of-flight magnetic resonance angiography
ADAPT: A direct aspiration first-pass technique
CMOSS: Carotid or middle cerebral artery occlusion surgery study
DM: Diabetes mellitus
IHD: Ischemic heart disease
HTN: Hypertension
MT: Mechanical thrombectomy
SWIM: Stent retriever partially retracted with intermediate catheter for mechanical thrombectomy
mRS: Modified Rankin scale
MRC: Medical research council
SAH: Subarachnoid hemorrhage
