# Supplementary figures and images for: Middle cerebral artery fenestration presenting with cerebral ischemia: a case report and review of the literature
Source: Front Med (Lausanne). 2026 Jun 9;13:1879070. doi: 10.3389/fmed.2026.1879070 (PMC13286832; doi:10.3389/fmed.2026.1879070)

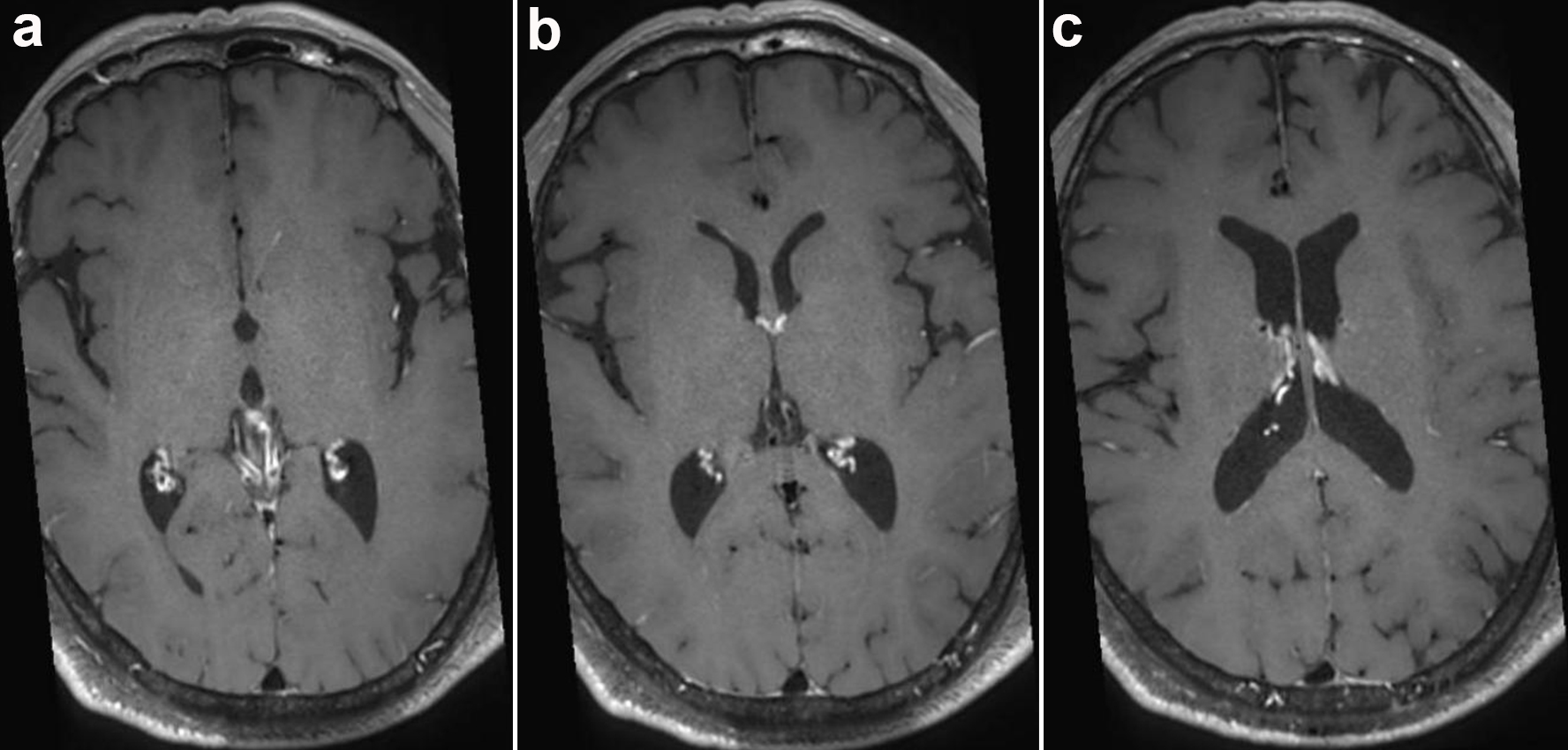

Supplement: Supplementary file 1 [file Image_1.JPEG]
