# Supplementary material for: Middle cerebral artery fenestration presenting with cerebral ischemia: a case report and review of the literature
Source: Front Med (Lausanne). 2026 Jun 9;13:1879070. doi: 10.3389/fmed.2026.1879070 (PMC13286832; doi:10.3389/fmed.2026.1879070)
Supplement: Supplementary file 2 [file Table_1.DOCX]

Figure S1. High-resolution vessel wall MRI (HRMR-VWI) showed no gyriform enhancement.
